# Late presentations and missed opportunities among newly diagnosed HIV patients presenting to a specialty clinic in Lebanon

**DOI:** 10.1038/s41598-024-55277-1

**Published:** 2024-04-09

**Authors:** Maya Mahmoud, Tala Ballouz, Chloe Lahoud, Jana Adnan, Paola Abi Habib, Reem Saab, Haya Farhat, Mohammad El Hussein, Nesrine Rizk

**Affiliations:** 1 Department of Internal Medicine, American University of Beirut Medical Center, Beirut, Lebanon; 2 Epidemiology, Biostatistics and Prevention Institute (EBPI), University of Zurich (UZH), Zurich, Switzerland; 3 Faculty of Medicine, American University of Beirut Medical Center, Beirut, Lebanon; 4 Division of Infectious Diseases, Department of Internal Medicine, American University of Beirut Medical Center, Riad El Solh, Beirut, 1107 2020 Lebanon

**Keywords:** HIV, AIDS, Late presenters, Missed opportunities, PLWH, AIDS-defining conditions, HIV infections, HIV infections

## Abstract

Late presentation to medical care of individuals infected with the human immunodeficiency virus (HIV) is linked to poor outcomes and increased morbidity and mortality. Missed opportunities for a prompt diagnosis are frequently reported among late presenters. We aimed to estimate the proportion of late presenters and missed opportunities in diagnosis among newly diagnosed HIV-positive subjects presenting to a specialty clinic in Lebanon. This is a retrospective chart review of all newly diagnosed adult HIV-positive subjects presenting to clinic from 2012 to 2022. Demographic, laboratory, and clinical data were collected at initial HIV diagnosis or presentation to medical care. We defined late presentation as having a CD4 count < 350 or AIDS-defining event regardless of CD4 count. Advanced disease is defined as having a CD4 count below 200 cells/μL or the presence of an AIDS-defining illness, regardless of the CD4 count. A missed opportunity was defined as the presence of an indicator condition (IC) that suggests infection with HIV/AIDS during 3 years preceding the actual HIV diagnosis and not followed by a recommendation for HIV testing. The proportions for demographic, epidemiological, and clinical characteristics are calculated by excluding cases with missing information from the denominator. Our cohort included 150 subjects (92.7% males; 63.6% men who have sex with men (MSM); 33.3% heterosexuals; median age 30.5 years at diagnosis). 77 (51.3%) were late presenters and 53 (35.3% of all subjects, 68.8% of late presenters) had advanced HIV on presentation. Up to 76.5% of late presenters had a presentation with an HIV-related condition at a healthcare provider without getting HIV test within the previous 3 years. The most frequent ICs were weight loss, generalized lymphadenopathy, constitutional symptoms, and chronic idiopathic diarrhea. Overall mortality rate was 4% (6/150 individuals). All-cause mortality among those who presented with AIDS was 15.4% (6/39 subjects). In our setting, late presentations and missed opportunities for HIV diagnosis are common. In the Middle East, AIDS mortality remains high with a large gap in HIV testing. To effectively influence policies, comprehensive analyses should focus on estimating the preventable health and financial burdens of late HIV presentations. Another concern pertains to healthcare providers’ attitudes and competencies.

## Introduction

Antiretroviral therapy (ART) remains one of the most important medical advancements in the twentieth century. There is ample evidence that effective ART improves cellular immunity and subsequently reduces AIDS-related morbidity and mortality. However, achieving the full benefits of ART is dependent on early HIV detection and initiation of treatment^1^. Late diagnosis of HIV has been associated with poorer health outcomes^2^, increased healthcare costs, and risk of onward transmission^3–7^. Yet, even in countries with adequate HIV testing recommendations and healthcare resources, late presenters (defined as those with a CD4 count less than 350 cells/mm^3^ or the presence of an AIDS-related illness at presentation) still constitute at least half of people living with HIV (PLWH)^8–11^ and continue to be a hurdle to HIV eradication efforts globally^12^.

Several sociodemographic, psychosocial, and structural risk factors—at the patient, provider, and policy level—have been identified to be associated with late presentation. Fear of HIV-related stigma and discrimination, poor social support, and low risk perception are among some of the common patient-related factors preventing people from seeking timely testing. Providers have described insufficient time and resources, the laborious process of counseling and consent, as well as low provider-perceived risk of transmission as barriers to offering an HIV test^13,14^. Studies of missed opportunities for earlier diagnoses have shown that individuals with late presentations had often presented to healthcare settings several times, sometimes with indicator conditions (ICs) before an HIV test was eventually made^15^. Meanwhile, the presence of punitive laws and policies, such as the criminalization of sex work and same-sex sexual acts, in some countries deter individuals from seeking HIV testing^16^.

While several studies have been conducted worldwide to investigate late presentations and missed opportunities, only a few have been conducted in the Middle East and North Africa (MENA) region^15,17–19^. Although the region has seen significant improvements in HIV services, early HIV diagnosis remains a challenge^7^. Recent numbers from the region show that only 67% of PLWH are aware of their status, and a considerable proportion of individuals newly diagnosed with HIV present with an advanced stage^20^. In Lebanon, the first HIV case was reported in 1984^21^, which has evolved to reach approximately 3000 cases in 2021^22^. The prevalence rate of HIV in Lebanon is less than 0.1%, with indications of a concentrated epidemic among marginalized populations, especially MSM. The prevalence of missed diagnoses and late presentations in Lebanon is unknown.

In this study, we aimed to (1) assess the epidemiologic characteristics of subjects presenting to an HIV clinic in an academic medical center in Lebanon, (2) examine the rate and risk factors of late presentations, and (3) quantify missed opportunities among late presenters.

## Materials and methods

Our study is a retrospective chart review of all newly diagnosed, treatment-naïve HIV-positive individuals, aged more than 18 years old, who presented to the American University of Beirut Medical Center (AUBMC) between January 1, 2012 and December 31, 2022. The AUBMC is an academic medical center in Lebanon with over 365 beds and a large outpatient department. The HIV-centered services started in 1984 and includes outpatient and inpatient services. The study was approved by the ethics committee, the Institutional Review Board (IRB) of the American University of Beirut Medical Center (AUBMC). The requirement for informed consent was waived by the Institutional Review Board at AUBMC due to the retrospective nature of the study. All research activities and methods were performed in accordance with the guidelines stated in the declaration of Helsinki and Belmont Report for research involving human subjects.

The subjects’ medical records were reviewed to collect demographic and clinical data including age at diagnosis, gender, nationality, sexual orientation, HIV transmission route, CD4 cell count, AIDS-defining conditions, and clinical indicator diseases at the time of diagnosis of HIV infection. MSM were defined as male participants reporting a homosexual or bisexual HIV-transmission mode and/or a sexual preference at the time of visit.

### Outcome definitions

The primary outcome of interest was the proportion of individuals with a late presentation, defined as presenting for care with a CD4 cell count below 350 cells/μL at HIV diagnosis, or presenting with an AIDS-defining event regardless of the CD4 cell count^23^. Secondary outcomes included (1) factors associated with late presentation, (2) the proportion of individuals presenting with advanced HIV disease (AHD), defined as a CD4 count below 200 cells/μL or the presence of an AIDS-defining illness regardless of CD4 count^24^, and (3) missed opportunities for diagnoses, defined as failure to diagnose HIV in the presence of an IC that should have triggered testing for HIV as per guidelines^25^. Indicator conditions (IC) are classified as “AIDS defining illnesses” events and “other events” that are known to be associated with advanced HIV but not categorized as AIDS-defining^26^. Any IC that was present in the 3 years preceding HIV diagnosis, and not followed by a recommendation for HIV testing was considered a missed opportunity for earlier HIV diagnosis. ICs documented within 1 month of HIV diagnosis were considered related to the newly diagnosed disease and therefore not considered as a missed opportunity.

### Statistical methods

We used descriptive statistics to analyze participant characteristics and outcomes of interest. Continuous variables are reported as median with interquartile range (IQR); categorical or ordinal variables as frequencies (N) and percentages (%). We explored the associations of several predictor variables with the outcome of late presentation using univariate and multivariable logistic regression model. Model selection was based on findings from other studies and age at diagnosis, gender, mode of transmission, and nationality were included. The calculated proportions for demographic, epidemiological, and clinical characteristics are derived after excluding cases with missing information from the denominator. We reported odds ratio (OR) with 95% confidence intervals (CI). All analyses were conducted in R (version 4.1, May 2021).

## Results

### Participant characteristics at diagnosis

A total of 150 individuals newly diagnosed with HIV presented to our clinic between 2012 and 2022. The median age was 30.5 years (IQR 26–42 years), and the majority identified as men (N = 139, 92.7%) and Lebanese (N = 119, 79.3%) (Table 1). Most of the non-Lebanese individuals were Arab nationals (primarily originating from Iraq, Syria and Saudi Arabia) presenting to Lebanon for medical care. Overall, 82 (63.6%) individuals acquired HIV through MSM contact and 43 (33.3%) through heterosexual contact. Only 3.1% (N = 4) of patients reported IV drug use as the mode of HIV transmission.

**Table 1:** Demographic, epidemiological and clinical characteristics of included participants at the diagnosis of HIV*.

Variable	Total participants (N = 150)
Age, median (IQR)	30.5 (26.0–42.0)
Age group in years	
< 25	33 (22.0%)
25–49	93 (62.0%)
≥ 50	24 (16.0%)
Gender, man N (%)	139 (92.7%)
Nationality, Lebanese N (%)	119 (79.3)
Highest education level	
Primary/middle school	2 (2.7%)
High school	11 (15.1%)
University or higher	60 (82.2%)
* Missing*	77
Employment	
Employed	68 (81.9%)
Unemployed	6 (7.2%)
Housewife	2 (2.4%)
Student	7 (8.4%)
* Missing*	67
Setting	
Urban	81 (90.0%)
Rural	9 (10.0%)
* Missing*	60
Risk category	
MSM	82 (63.6%)
Heterosexual	43 (33.3%)
Intravenous drug use	4 (3.1%)
* Missing*	21
CD4 cell count at diagnosis, median	331.0 (164.5–498.5)
< 200	41 (29.5%)
200–350	34 (24.5%)
> 350	64 (46.0%)
* Missing*	11

*The proportions provided are based solely on available information, and any missing data is not taken into account in the calculations.

Twenty-four (16%) of our newly diagnosed patients were aged more than 50 years old. Among them, 23 were males, and 1 was female. Within this sub-group, the median age at diagnosis was 58 years old. The median CD4 count was 197 cells/mm^3^, compared to 353 in our patients aged less than 50 years, with 13 (54.2%) patients presenting with a CD4 count less than 200. Fifteen were heterosexuals, and nine were men who have sex with men (Supplementary Table 1).

### Late presentation

Overall, 77 individuals (51.3%) were late presenters and had a CD4 cell count of < 350 cells/mm^3^ at the time of HIV diagnosis. Among those, 43 (55.8%) had a CD4 cell count of < 200 cells/mm^3^ and 39 (50.6%) presented with AIDS-related conditions. A total of six individuals out of 150 died (15.4% of those presenting with an AIDS defining illness, 4.0% of all participants). The median CD4 cell count at HIV diagnosis was 506.5 (436.2–638.8) and 191.0 (67.0–258.0) cells/mm3 in non-late presenters and late presenters, respectively.

Late presentation was significantly associated with older age (OR 1.05, 95% CI 1.02–1.09, p = 0.003). Although an association with MSM transmission was observed, it did not reach statistical significance (OR 2.47, 95% CI 0.98–6.66, p = 0.062) (Supplementary Table 2).

### Missed opportunities for earlier HIV testing

To identify indicator conditions, we reviewed medical records before the presentation and HIV diagnosis. Comprehensive data on indicator conditions were present in 51 of 77 charts of late presenters (66.2%). In total, there were 68 ICs among 39 participants (76.5%) in the preceding 3 years prior to HIV testing. Of the 39 participants with a missed opportunity for HIV diagnosis, 27 (69.2%) subjects had one or more AIDS-defining conditions and 9 (23.1%) subjects had ICs consistent with AIDS defining conditions. The most frequent ICs were unexplained weight loss (18/68, 26.5%), unexplained lymphadenopathy (9/68, 13.2%) unexplained fatigue and malaise (7/68, 10.3%), unexplained chronic diarrhea (6/68, 8.8%) and unexplained fever with no apparent etiology (6/68, 8.8%). Seven AIDS-defining ICs were identified. Those included recurrent pneumonia in five cases, four of which were confirmed to be *pneumocystis jirovecii* pneumonia (PCP) (Table 2).

**Table 2:** Indicator conditions associated with missed opportunity for HIV diagnosis.

Indicator conditions	n = 68 (%)
General symptoms	
Unexplained weight loss	18 (26.5%)
Unexplained fatigue and malaise	7 (10.3%)
Unexplained chronic diarrhea	6 (8.8%)
Unexplained fever	6 (8.8%)
Chronic headache	2 (2.9%)
Recurrent bacterial and viral infections	
Community-acquired pneumonia	5 (7.4%)
Brucellosis	2 (2.9%)
Cytomegalovirus colitis*	1 (1.5%)
Mucocutaneous symptoms	
Unexplained lymphadenopathy	9 (13.2%)
Varicella Zoster	1 (1.5%)
Herpes simplex, ulcer*	1 (1.5%)
Exanthema	2 (2.9%)
Diseases associated with HIV infection	
Hepatitis B or C	2 (2.9%)
Sexually transmitted infections	2 (2.9%)
Candidiasis, esophageal*	2 (2.9%)
Cerebral toxoplasmosis*	1 (1.5%)
Non-Hodgkin lymphoma*	1 (1.5%)

*Indicates indicator conditions (IC) which are AIDS defining.

### Late presenters with advanced HIV

Among the 77 late presenters, 53 (68.8% of late presenters and 35.3% of all newly diagnosed) presented with an advanced HIV stage. Of these 53 participants, 39 (73.6%) had at least one AIDS defining illness at the time of diagnosis (44 conditions in total). The most frequent presentations were HIV wasting (16/44, 36.4%), PCP (9/44, 20.5%), candida esophagitis (4/44, 9.1%), cerebral toxoplasmosis (3/44, 6.8%), mycobacterium tuberculosis infection (2/44, 4.5%), Kaposi sarcoma (2/44, 4.5%), and Burkitt lymphoma (2/44, 4.5%) (Table 3).

**Table 3:** AIDS defining conditions at presentation among late presenters.

AIDS defining condition	N = 44*
HIV wasting	16 (36.4%)
*Pneumocystis jirovecii* pneumonia	9 (20.5%)
Candida esophagitis	4 (9.1%)
Cerebral toxoplasmosis	3 (6.8%)
Mycobacterium tuberculosis	2 (4.5%)
Burkitt lymphoma	2 (4.5%)
Kaposi’s sarcoma	2 (4.5%)
Cryptococcus meningitis	1 (2.3%)
Chronic herpes simplex ulcer	1 (2.3%)
Salmonella septicemia, recurrent	1 (2.3%)
Cytomegalovirus pneumonia	1 (2.3%)
Cytomegalovirus colitis	1 (2.3%)
Pneumonia, recurrent	1 (2.3%)

*Among 39 participants.

## Discussion

### Key findings

To the best of our knowledge, this is one of few studies in the MENA region assessing late presentations of HIV and missed opportunities for earlier diagnosis^17–19,27,28^. We found that more than half of the newly diagnosed subjects in our cohort (51.3%) were late presenters and 35.3% had advanced HIV disease on presentation. Mortality from HIV-related death was around 5% among our cohort while mortality from HIV in the world is approximately 2%^29^. Mortality among those presenting with AIDS in our cohort was approximately 16%. Almost three in four of late presenters had attended a medical facility for an IC in the 3 years preceding diagnosis; of these, almost one in four presented with an AIDS defining conditions without getting tested for HIV.

### Evidence in context

As of December 2022, Lebanon had an estimated 2600 PLWH, with an incidence rate below 0.03%^30^. It is important to note that reported numbers likely underestimate the true count of PLWH in Lebanon, primarily due to reliance on passive reporting. Our findings correspond with those presented in the national report. In fact, the 2018 UNAIDS report revealed that 26% of individuals newly diagnosed with HIV in Lebanon presented at an advanced stage, characterized by an initial CD4 count below 200 cells/mm^3^^31^. Few studies have described late presentations in the MENA region^32,33^. Our results are in line with data from Turkey and Iran. Studies conducted in Turkey found that 50–69% of the PLWH presented late to medical care, and 25–40% of subjects had advanced HIV at the time of diagnosis^26,34–37^. Similarly, a large retrospective cohort study conducted in Iran revealed a prevalence of late diagnosis in around 58.2% of subjects^17^. Surveillance studies from Yemen and Saudi Arabia showed higher prevalence of late HIV. The cohort study from Yemen showed that 83% of PLWH presented with a CD4 less than 350 and 52% with CD4 count less than 200^18^. The study from Saudi Arabia included 977 subjects and revealed that 20% of HIV positive subjects had a CD4 < 350 at diagnosis, and 50% presented with AIDS at diagnosis^19^. Late diagnosis indicates a gap in HIV testing^38,39^, which is a notable observation from the countries of the MENA region. In fact, according to the UNAIDS, by the end of 2018, more than half of PLWH in the MENA region were not aware of their seropositivity status^40^.

In our study, subjects who presented late were older and were men who had sex with men. Interestingly, women only represented 7.3% of our population (11 out of 150), indicating potential additional social obstacles that women encounter when seeking HIV care. This aligns with national data from Lebanon, indicating that the country faces a concentrated HIV epidemic among MSM, comprising 12% of cases^41^. While our study did not specifically address barriers to testing, the increased prevalence of late presenters among individuals aged more than 50 years and MSM in our cohort may be attributed to persistent barriers to adequate HIV testing^39^. This phenomenon could be linked to lower testing rates in these demographics, potentially influenced by social, cultural and legal barriers such as criminalization of homosexuality, stigma preventing adequate sexual education, lack of access to HIV testing and poor comprehensive sexual and reproductive health provision^42^. Around six out of ten people with HIV are from marginalized groups, including MSM, transgender individuals, IV drug users, sex workers, and their clients^43^. However, it is precisely these marginalized communities who encounter significant challenges in accessing HIV prevention, testing, treatment, and care services due to stigma and discrimination. We performed subgroup analyses for the subgroups late presenters with and without advanced disease (presented in Supplemental Table 3). As expected, the only difference was the CD4 count, 281 and 89 cells/mml for the without and with advanced disease, respectively.

PLWH in Lebanon continue to face social stigmatization and discrimination impacting different aspects of their lives. Particularly, the MSM population experiences homophobia and legal consequences, given that the Lebanese penal code prohibits sexual relations deemed "contradicting the laws of nature", punishable by up to a year in prison. Nevertheless, Lebanon is relatively more accepting of sexual rights compared to other countries in the MENA region, making it a favorable location for getting tested and treated for HIV^44^. HIV testing is available at medical laboratories, hospitals, or free of charge at Voluntary Counseling and Testing (VCT) centers in Lebanon. These centers are spread throughout the country, ensuring accessibility for the entire population, including refugees. Lebanon follows a comprehensive "treatment for all" strategy in addressing HIV/AIDS^45^. The Ministry of Public Health (MOPH) provides free treatment to over 60% of individuals aware of their HIV status including Syrian and Palestinian refugees.

There is a paucity of published data on missed opportunities in the MENA region. Similar to our findings, a study from Morocco reported that 69% of their 650-subject cohort had missed opportunities for HIV testing^15^. In contrast, studies from countries outside the MENA region such as Italy, Sweden, Germany and UK showed that 21–27% of newly diagnosed HIV subjects who sought medical care for ICs were not offered HIV testing^46–50^. The missed opportunity proportion is higher in our cohort. Limited awareness or knowledge among healthcare workers, along with negative perceptions and stigma associated with HIV within this group, may account for missed opportunities. Risk factors for HIV infection might not be adequately addressed by the treating physician. Firstly, subjects may not have disclosed their sexual activity, sexual orientation, and gender identity because of fear of discrimination and stigma. Secondly, healthcare workers with negative perceptions towards specific populations—sex workers, IV drug users, LGBTQ + community- and lack of adequate training regarding sexual health matters often fail to properly address the behaviors and sexual orientations of their subjects^51^.

Missed opportunities can lead to late detection and diagnosis of HIV with consequent associated complications including higher morbidity and mortality, altered response to antiretroviral therapy (ART)**,** increased cost of medical care, and HIV transmission within the community^46,52,53^. More efforts are needed to provide HIV-specific training and to eliminate stigma and discrimination related to HIV among healthcare providers.

## Limitations

Our study has several limitations that may have influenced our findings. Firstly, being a single-center study could restrict the generalizability of our results to the broader Lebanese population or other populations. The retrospective nature of our study also posed limitations on data collection, particularly regarding socioeconomic aspects such as housing situation, poverty, and risky sexual practices, which could have offered additional insights into factors associated with late presentation and missed opportunities.

Moreover, there is a potential underestimation of the proportion of missed opportunities in our population. Our results rely on data collected from medical records, and other opportunities may have been present but not documented. Conversely, we cannot guarantee that verbal recommendations for HIV testing by healthcare providers were documented or, if refused by the subject, leading to a possible overestimation of missed opportunities.

The collected data also lacked crucial clinical details on management and follow-up. Notably, some subjects were discharged to home with hospice care, despite their initial diagnosis being conducted at our center. The initiation of Antiretroviral Therapy (ART) presents an intriguing aspect; however, our data collection did not encompass this specific information for all subjects. Similarly, details regarding the time to death and potential Immune Reconstitution Inflammatory Syndrome (IRIS) were not included in our data collection.

Unforeseen circumstances significantly impacted our study, especially after 2019, affecting clinic follow-up, detailed history, and thorough evaluation and diagnostic investigation. Lebanon faced political turmoil and economic failure starting in 2019, resulting in disruptions to clinical operations and ongoing follow-up. The subsequent COVID-19 pandemic further compounded the situation by imposing additional movement restrictions through lockdowns, leading to several subjects either being lost to follow-up or conducting virtual visits.

## Conclusion

In our cohort, and likely in the MENA region, late presentation with HIV and missed opportunities for HIV diagnosis are common, even in instances where HIV testing is clearly indicated. To effectively influence policies, it is imperative to expand research efforts and conduct comprehensive analyses to quantify the proportion of late presenters and missed opportunities in the region, and to explore the factors contributing to these findings. Future studies should prioritize the estimation of the preventable financial burden associated with late HIV presentation resulting from diminished productivity and increased healthcare expenditure. Another concern pertaining to healthcare providers’ attitudes and competencies should trigger a serious reform in the healthcare provider curricula regarding sexual health and reproductive health issues.

### Supplementary Information

Supplementary Tables.

## Data Availability

De-identified participant data that underlie the results reported in this article can be shared upon reasonable requests to the corresponding author. Data requestors will need to sign a data access agreement form.
