# Supplementary material for: Late presentations and missed opportunities among newly diagnosed HIV patients presenting to a specialty clinic in Lebanon
Source: Sci Rep. 2024 Apr 9;14:8296. doi: 10.1038/s41598-024-55277-1 (PMC11004126; doi:10.1038/s41598-024-55277-1)
Supplement: Supplementary file 1 — Supplementary Tables. [file 41598_2024_55277_MOESM1_ESM.pdf]

SUPPLEMENTARY MATERIAL

Supplementary Table 1. Characteristics of Patients under and above 50 years old

		<50 years old (n= 126)		>50 years old (n=24)	
		Median (IQR)	N (%)	Median (IQR)	N (%)
Age at diagnosis		29 (25 to 34)		58 (55 to 60)	
Gender	Female		10 (7.9)		1 (4.2)
	Male		116 (92.1)		23 (95.8)
CD4 count		353 (199 to 512)		197 (67 to 335)	
CD4 group	Missing		9 (7.1)		0 (0)
	<200		30 (23.8)		13 (54.2)
	>350		60 (47.6)		4 (16.7)
	200-350		27 (21.4)		7 (29.2)
Sexual orientation	Bisexual		11 (8.7)		0 (0)
	Heterosexual		33 (26.2)		15 (62.5)
	MSM		82 (65.1)		9 (37.5)
Education	Missing		63 (50)		14 (58.3)
	High school		9 (7.1)		2 (8.3)
	Middle school		2 (1.6)		0 (0)
	University/higher		52 (41.3)		8 (33.3)
Employment	Missing		53 (42.1)		14 (58.3)
	Employed		60 (44.7)		8 (33.3)
	Housewife		2 (1.6)		0 (0)
	Student		7 (5.6)		0 (0)
	Unemployed		4 (3.2)		2 (8.3)

Supplementary Table 2. Associations of characteristics of late presenters at diagnosis with late presentation based on univariate and multivariable logistic regression analyses.

Characteristic		Univariate		Multivariable	
	N	OR (95 % CI)	p-value	OR	p-value
**Age at diagnosis (years)**	145	1.04 (1.01 to 1.07)	0.008	1.05 (1.02 to 1.09)	0.003
**Gender**	145				
Female		Ref.		Ref.	
Male		0.94 (0.36 to 5.97)	>0.90	0.58 (0.13 to 2.54)	0.5
**Transmission category**	145				
Heterosexual		Ref.		Ref.	
MSM		1.28 (0.62 to 2.65)	0.5	2.47 (0.98 to 6.66)	0.062
IDU		0.35 (0.02 to 2.97)	0.4	0.75 (0.03 to 7.28)	0.8
**Nationality**	145				
Lebanese		Ref.		Ref.	
Non-Lebanese		0.93 (0.42 to 2.07)	0.9	0.98 (0.40 to 2.40)	>0.90

Legend: OR= odds ratio, CI= confidence interval, IDU= intravenous drug use, MSM: men who have sex with men, N= number

Supplementary Table 3. Characteristics of Late Presenters with and without Advanced Disease.

		Late presenters without advanced disease (n=24)		Late presenters with advanced disease (n=53)	
		Median (IQR)	N (%)	Median (IQR)	N (%)
Age at diagnosis		28 (25 to 33)		40 (30 to 55)	
Gender	Female		1 (4.2)		5 (9.4)
	Male		23 (95.8)		48 (90.6)
Nationality	Lebanese		19 (79)		42 (79.2)
	non-Lebanese		5 (21)		11 (20.8)
CD4 count		281.68 (251.5 to 330)		89.00 (27.7 to 195.1)	
CD4 group	<200		0 (0)		43 (81.1)
	200 -350		23 (95.8)		9 (17)
	>350		1 (4.2)		1 (1.9)
Sexual Orientation	Bisexual		4 (16.7)		2 (3.8)
	Heterosexual		4 (16.7)		18 (34)
	MSM		16 (66.7)		33 (62.3)
Risk Category	IV drug use		3 (12.5)		1 (1.9)
	MSM		16 (66.6)		33 (62.3)
	Heterosexual		4 (16.6)		18 (34)
	Missing		1 (4.2)		1 (1.8)
Death*	Yes		0 (0)		6 (11.3)
	No		24 (100)		47 (88.7)
	Death cause (n= 6)				
Death cause	Kaposi Sarcoma				2 (33.3)
	HIV wasting				2 (33.3)
	CNS Toxoplasmosis				1 (16.6)
	Tuberculosis				1 (16.6)
